# Functional preoperative assessment of coronal knee laxity better predicts postoperative patient outcomes than intraoperative surgeon‐defined laxity in total knee arthroplasty

**DOI:** 10.1002/ksa.12400

**Published:** 2024-09-03

**Authors:** Ishaan Jagota, Rami M. A. Al‐Dirini, Mark Taylor, Joshua Twiggs, Brad Miles, David Liu

**Affiliations:** ^1^ Research and Development 360 Med Care Sydney Australia; ^2^ Research and Development Enovis ANZ Sydney Australia; ^3^ College of Science and Engineering Flinders University Adelaide Australia; ^4^ The Gold Coast Centre for Bone and Joint Surgery Palm Beach Queensland Australia

**Keywords:** balance, coronal knee laxity, preoperative planning, stress radiograph, total knee arthroplasty

## Abstract

**Purpose:**

Intraoperative laxity assessments in total knee arthroplasty (TKA) are subjective, with few studies comparing against standardised preoperative and postoperative assessments. This study compares coronal knee laxity in TKA patients awake and anaesthetised, preprosthesis and postprosthesis implantation, evaluating relationships to patient‐reported outcome measures.

**Methods:**

A retrospective analysis of 49 TKA joints included preoperative and postoperative computed tomography scans, stress radiographs and knee injury and osteoarthritis outcome score (KOOS) questionnaire results preoperatively and 12 months postoperatively. The imaging was used to assess functional laxity (FL) in awake patients, whereas computer navigation measured intraoperative surgical laxity (SL) preimplantation and postimplantation, with patients anaesthetised. Varus and valgus stress states and their difference, joint laxity, were measured.

**Results:**

SL was greater than FL in both preimplantation [8.1° (interquartile range, IQR 2.0°) and 3.8° (IQR 2.9°), respectively] and postimplantation [3.5° (IQR 2.3°) and 2.5° (IQR 2.7°), respectively]. Preimplantation, SL was more likely than FL to categorise knees as correctable to ±3° of the mechanical axis. Preoperative FL correlated with KOOS Symptoms (*r* = 0.33, *p* = .02) and quality of life (QOL) (*r* = 0.38, *p* = .01), whereas reducing medial laxity with TKA enhanced postoperative QOL outcomes (*p* = .02).

**Conclusions:**

Functional coronal knee laxity assessment of awake patients is generally lower than intraoperative surgical assessments of anaesthetised patients. Preoperative SL may result in overcorrection of coronal TKA alignment, whereas preoperative FL better predicts postoperative patient outcomes and reflects the patients' native and tolerable knee laxity. Preoperative FL assessment can be used to guide surgical planning.

**Level of Evidence:**

Level II.

**Abbreviations**
2D: two‐dimensional
3D: three‐dimensional
ADL: activities of daily living
CT: computed tomography
FL: functional laxity
HKA: hip–knee–ankle
IQR: interquartile range
KOOS: knee injury and osteoarthritis outcome score
OA: osteoarthritis
Post‐FL: postimplantation functional laxity
Post‐SL: postimplantation surgical laxity
Pre‐FL: preimplantation functional laxity
Pre‐SL: preimplantation surgical laxity
PROM: patient‐reported outcome measure
QOL: quality of life
ROM: range of motion
SL: surgical laxity
TKA: total knee arthroplasty

## INTRODUCTION

In the pursuit of improved satisfaction rates in total knee arthroplasty (TKA), surgical technique and prostheses designs have evolved to better emulate the native knee anatomy and kinematics [3, 32, 33]. Recent advances in the understanding of knee phenotypes, such as the functional knee phenotypes [11] and the coronal plane alignment of the knee [25], allow estimation of the native coronal bony anatomy of osteoarthritic knees [10]. Although such phenotypes display some relationships to coronal knee laxity, there remains high variability in ligamentous knee morphology [6, 7, 12]. An appropriate assessment of the native coronal knee laxity is required for TKA planning to optimise ligament balance with minimal disruption to the soft‐tissue envelope during TKA [30, 33]. However, the current surgical standard for native coronal knee laxity assessment involves intraoperative assessment before bone resections by applying manual varus and valgus forces with the patient anaesthetised. This method for native knee laxity assessment lacks standardisation and remains subjective due to nonuniform manual force application. Further, surgeon assessment of balance has been reported to be inconsistent and influenced by surgeon experience [24, 38, 48], thus raising concerns about its suitability to represent the native or constitutional knee laxity.

Alternate noninvasive methods to preoperatively assess patient‐specific coronal knee laxity using X‐rays under varus and valgus loading have been proposed [14, 41]. These techniques offer objective laxity assessment, are predictive of the extent of tissue release [21], and importantly, profile native coronal knee laxity with the patient awake. This approach aids soft‐tissue‐informed preoperative TKA planning, and enables systematic comparisons of coronal laxity before and after surgery, enhancing understanding of its impact on patient outcomes. Prior research has examined the difference in joint laxity between timepoints, with patients awake and anaesthetised [2, 14, 41] and analysed the relationship between joint laxity at a single timepoint, such as postoperatively and patient‐reported outcomes measures (PROMs) [9, 18, 28]. However, the relationship between changes in coronal laxity across operative states and PROMs remains underexplored.

The primary aim of this study is to compare coronal knee laxity of a TKA cohort preoperatively and postoperatively when awake, using a standardised protocol and intraoperatively preprosthesis and postprosthesis implantation using the surgical standard approach with the patient anaesthetised. Secondary aims include investigating the relationship between both knee laxity and the difference in laxity between operative states and PROMs. We hypothesise that preprosthesis and postprosthesis implantation laxities are lower in patients when awake compared with anaesthetised, and the difference between awake and anaesthetised laxity will be lower postimplantation than preimplantation. Further, we hypothesise that awake laxity assessments will have stronger relationships to PROMs than anaesthetised intraoperative assessments.

## MATERIALS AND METHODS

**Figure 1:**
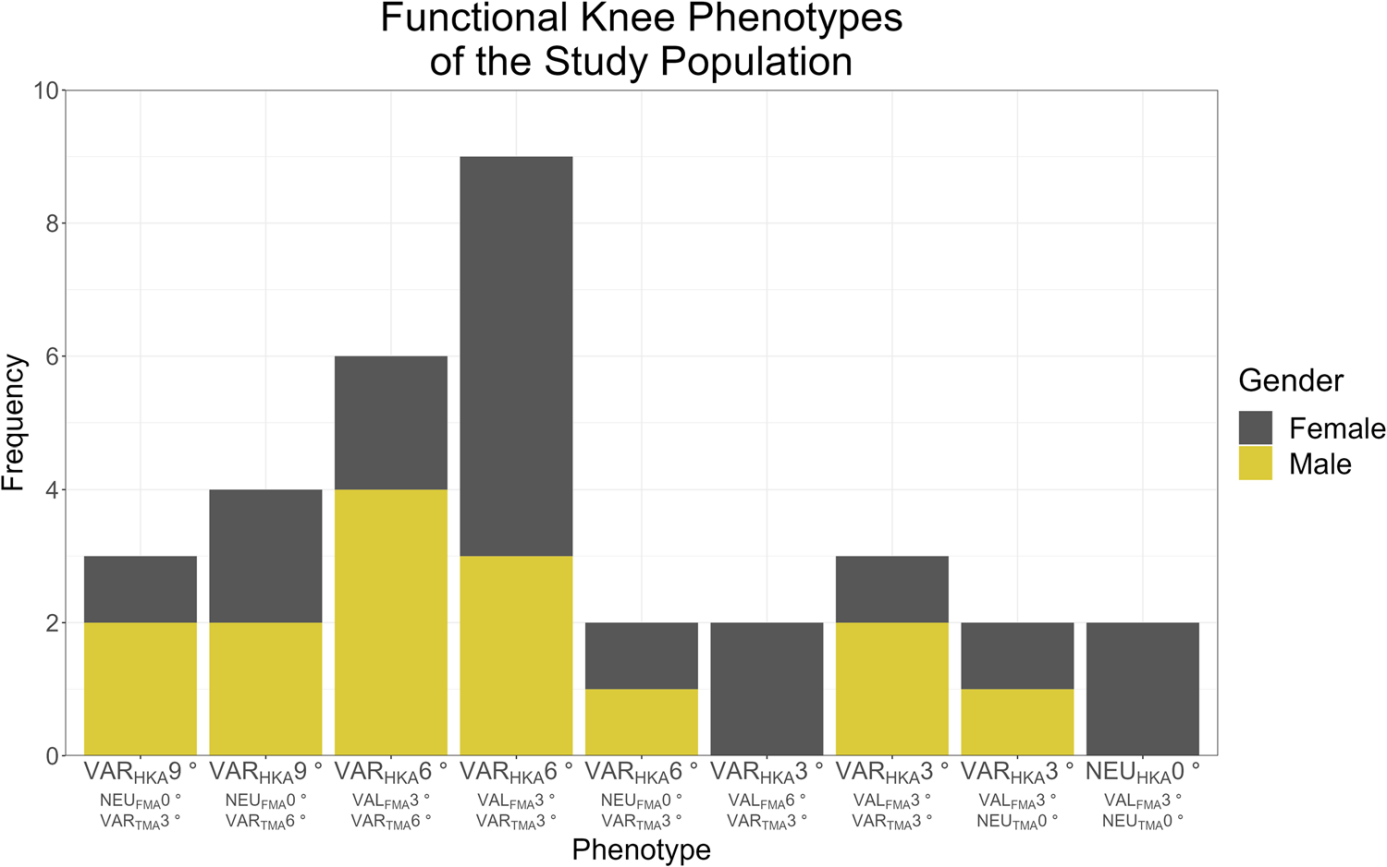
Common functional knee phenotypes observed within the study population. These nine phenotypes contain over 67% (33) of the study population, with the remaining 16 knees all spread across different phenotypes. FMA, femoral mechanical angle; HKA, hip–knee–ankle angle; NEU, neutral; TMA, tibial mechanical angle; VAL, valgus; VAR, varus.

A retrospective analysis was performed on 49 joints from 46 patients undergoing primary TKA for knee osteoarthritis (OA), recruited by one surgeon between 29 February 2016 and 29 March 2017. Patients with inflammatory arthritis, extra‐articular deformity, undergoing complex primary or unicompartmental to TKA, or those unable to complete imaging due to contraindications, refusal or lack of access, were excluded from the study. A preoperative analysis of functional knee phenotypes [11] was used to describe the coronal knee anatomy of the study population (Figure 1). A power analysis with 80% power, a 5% two‐tailed significance level and an assumption of no attrition determined a minimum sample size of 30 subjects. Ethics approval was granted by the Bellberry Human Research Ethics Committee, application number 2012‐03‐710.

### PROMs

Patients completed the knee injury and osteoarthritis outcome score (KOOS) questionnaire preoperatively and 12 months postoperatively. The five KOOS subscales of pain, symptoms, activities of daily living (ADL), quality of life (QOL) and sports and improvement in score from preoperative to postoperative were calculated.

### Surgical technique and intraoperative laxity assessment

All TKAs were performed by the investigating surgeon using a Corin Omni Apex cruciate‐retaining prosthesis. Intraoperatively, with the patient anaesthetised, ligament laxity was measured using the Corin OMNINav computer navigation system at two timepoints; preimplantation, before bone preparation, osteophyte removal and soft‐tissue release except for medial arthrotomy for joint exposure and landmarking using the navigation tool; and after prosthesis implantation, before joint closure. Varus and valgus stresses were manually applied throughout the range of motion (ROM) and the OMNINav system recorded the resulting hip–knee–ankle (HKA) angles. The stressed HKA angle measurements at 10° flexion were extracted. OMNINav was also used for the delivery of tibial and femoral resections and gap balancing. The surgeon targeted a mechanically aligned TKA within ±3° of neutral to the mechanical axis. No routine medial release was performed initially except to access and remove significant osteophytes. The tibia cut was performed first neutral to the mechanical axis. The extension and flexion (90°) gaps were then retensioned using a laminar spreader to plan the final femoral component alignment, aiming for equal medial and lateral gaps and 1‐mm greater flexion than the extension gap. The deep medial collateral ligament was released if necessary to remove medial tibial osteophytes. A medial capsular release was performed along the tibial cut rim if the overall HKA was greater than 3° varus but was not required in any cases in the study cohort. Any residual fixed flexion beyond 5° was corrected with posterior capsular release from the femur.

### Preoperative and postoperative radiology capture and image processing

Within 2 months preoperatively, patients underwent a long‐leg supine computed tomography (CT) scan as per the AURORA protocol [45] and two radiographs under varus and valgus stress using a Telos SD‐900 device. The device features two countersupports on one side of the leg and a centrally positioned force pad on the other. During varus and valgus stress, the force pad was placed medially and laterally to the knee, respectively, aligning with the tibial tuberosity. Countersupports were positioned at the proximal femur and distal tibia and with the knee flexed between 0° and 20° [13, 23, 46]. The operator gradually increased the applied force to a maximum of 150 N [13, 23, 46], stopping if the patient indicated significant discomfort. Radiographs were then taken. A subset of 37 patients also received 6‐month postoperative imaging, following the same protocol. All imaging was performed at a single radiology centre by specifically trained technicians.

**Figure 2:**
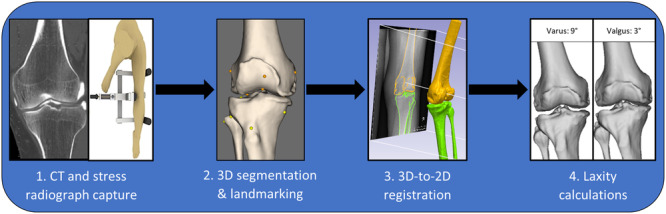
Summary of the preoperative and postoperative laxity assessment workflow, which involves computed tomography (CT) and stress radiograph capture, three‐dimensional (3D) segmentation and landmarking, 3D‐to‐two‐dimensional (2D) registration and the calculation of laxity measurements.

Preoperative CT scans were segmented and landmarked (Supporting Information S1: Appendix A) using Simpleware ScanIP (version M‐2017.06, Synopsys, Inc.), producing three‐dimensional (3D) bone models. These models were registered to the two‐dimensional (2D) stress radiographs using Mimics (Materialise) R19 Research software following Li et al.'s method [23], allowing HKA angle measurements at varus and valgus extents. Postoperatively, prostheses and preoperative bone models were registered to the postoperative CT scan via 3D‐to‐2D registration using Simpleware ScanIP, yielding postoperative 3D bone models and landmarks. These were then registered to the postoperative stress radiographs using the established preoperative process. The imaging workflow is summarised in Figure 2. Both preoperatively and postoperative segmentation, landmarking and registrations were performed by an engineer and were quality checked by a senior engineer. All radiographic measurements were calculated using a custom script in Posit R Studio v1.2.5019, using the anatomical landmarks in the CT and stressed positions. These processes have reported submillimetre and subdegree intraobserver and interobserver variability and high reliability [5, 45].

### Laxity measurements

Table 1 outlines the anatomical measurements employed in our study. HKA angles under varus and valgus stress in the preoperative and postoperative and preimplantation and postimplantation (intraoperative) settings were used to calculate knee laxity. Larger laxity values indicated looser knees; smaller values indicated tighter knees. The OMNINav system's HKA angles under stress determined the surgical laxity (SL) both preimplantation (Pre‐SL) and postimplantation (Post‐SL), with patients anaesthetised. Functional laxity (FL) describes knee laxity in conscious patients with the standardised force application both preoperatively (Pre‐FL) and postoperatively (Post‐FL). Differences in stress HKA angles and joint laxity across operative states were also calculated.

**Table 1:** Description and formulae of key anatomical measurements performed.

Term	Description	Formula
HKA angle	Angle subtended by the femoral and tibial mechanical axes. A positive angle denotes a varus HKA angle. Measured for the preoperative and postoperative supine CT positions. Measured for the varus and valgus stress positions preoperatively and postoperatively as well as intraoperatively, both preimplantation and postimplantation.	
Laxity	The absolute difference between the varus and valgus stress angles. Measured preoperatively and postoperatively as well as intraoperatively, both preimplantation and postimplantation.	Laxity = |HKA_Varus_–HKA_Valgus_|
SL	Knee laxity as measured intraoperatively with the patient anaesthetised. Measured preimplantation (Pre‐SL) and postimplantation (Post‐SL).	
FL	Knee laxity as measured with the patient in a conscious state. Measured preoperatively (Pre‐FL) and postoperatively (Post‐FL).	
Joint laxity difference	Difference in laxity between two operative states. Determined between the surgical measurements, functional measurements, before implantation and after implantation.	Preimplantation: Pre‐SL–Pre‐FL Postimplantation: Post‐SL–Post‐FL Surgical: Pre‐SL–Post‐SL Functional: Pre‐FL–Post‐FL
Correctability	Indicates whether the laxity range falls within ±3° of neutral to the mechanical axis.	

Abbreviations: CT, computed tomography; FL, functional laxity; HKA, hip–knee–ankle; SL, surgical laxity.

### Statistical analysis

Patient demographics were assessed for mean and standard deviation. Pearson's linear correlations assessed relationships between each pair of laxity measurements and between anatomical measurements (joint laxity, varus and valgus stress HKA angles) and patient outcomes (preoperative, postoperative and improvement of the five KOOS subdomain scores). Patients were also categorised by whether they displayed greater joint laxity and stress HKA angles, either preoperatively or preimplantation and subsequently PROMs were evaluated using the Mann–Whitney *U* test. Similar analyses were performed comparing PROMs between the postoperative and postimplantation, preoperative and postoperative and preimplantation and postimplantation settings. A *p* value of .05 indicated statistical significance and all statistical analyses were performed using Posit R Studio v1.2.5019.

## RESULTS

Table 2 summarises patient demographics. Postimplantation and postoperative data were available for 44 and 37 of the 49 joints, respectively. There was a significant difference between the mean preoperative (5.1°, interquartile range [IQR] 3.9°) and postoperative (2.2°, IQR 2.2°) supine CT HKA angles (*p* < .001).

**Table 2:** Patient demographics.

Variables	Total	Men	Women
Joints	49	23	26
Left/right	19/30	10/13	9/17
Age (years)	67.3 (IQR 9.9)	69.4 (IQR 8.9)	65.3 (IQR 12.0)
Preoperative CT HKA (°, Varus)	5.1 (IQR 3.9)	5.9 (IQR 3.6)	4.3 (IQR 3.7)
Postoperative CT HKA (°, Varus)	2.2 (IQR 1.8)	2.3 (IQR 2.0)	2.1 (IQR 1.6)

Abbreviations: CT, computed tomography; HKA, hip–knee–ankle angle; IQR, interquartile range.

**Figure 3:**
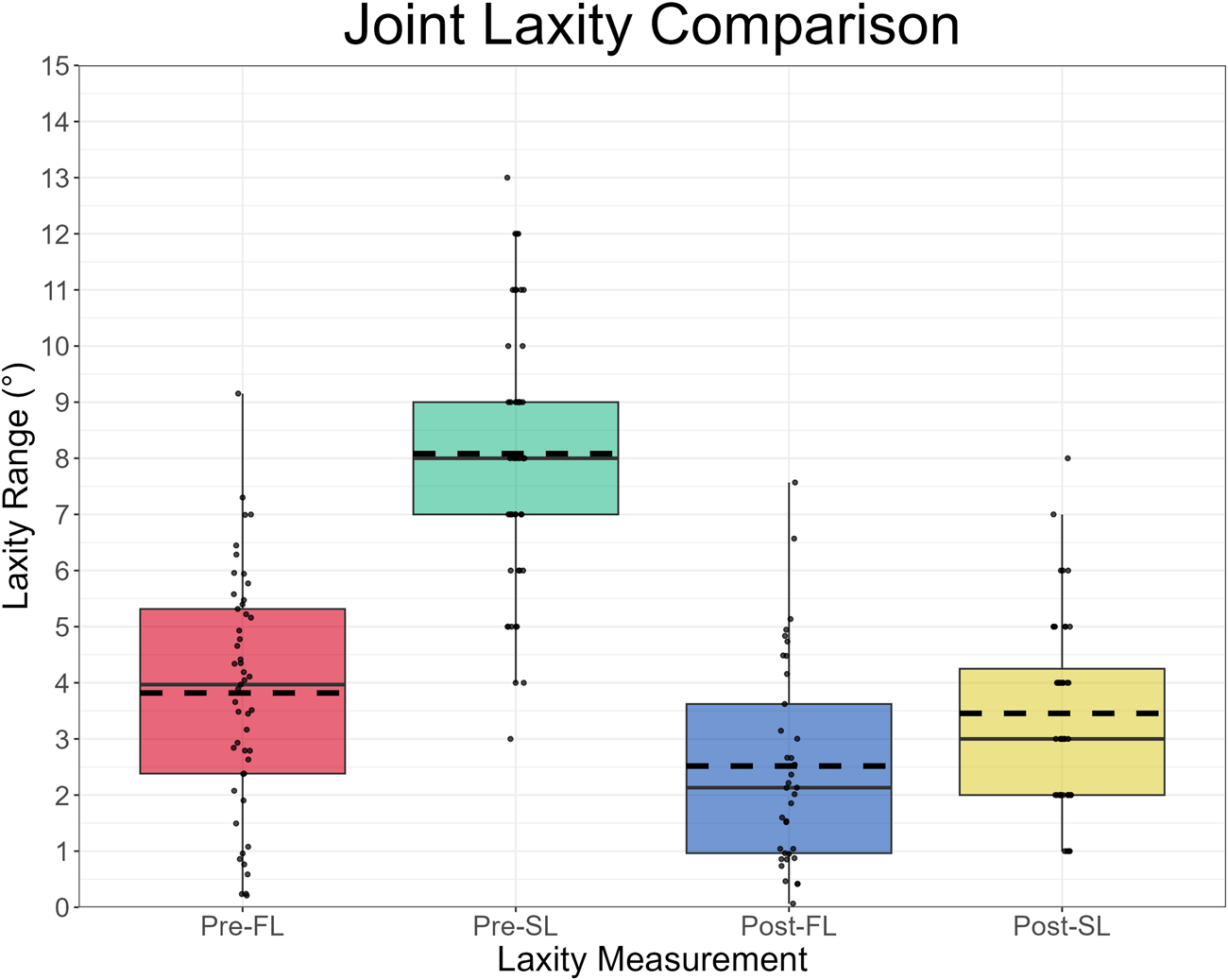
Boxplot of the laxity measurements across the preoperative, intraoperative and postoperative settings. Each pair of measurements (except preoperative functional laxity [Pre‐FL] and postimplantation surgical laxity [Post‐SL]) was statistically significantly different to each other (*p* < .05). Pre‐SL, preimplantation surgical laxity; Post‐FL, postoperative functional laxity.

**Figure 4:**
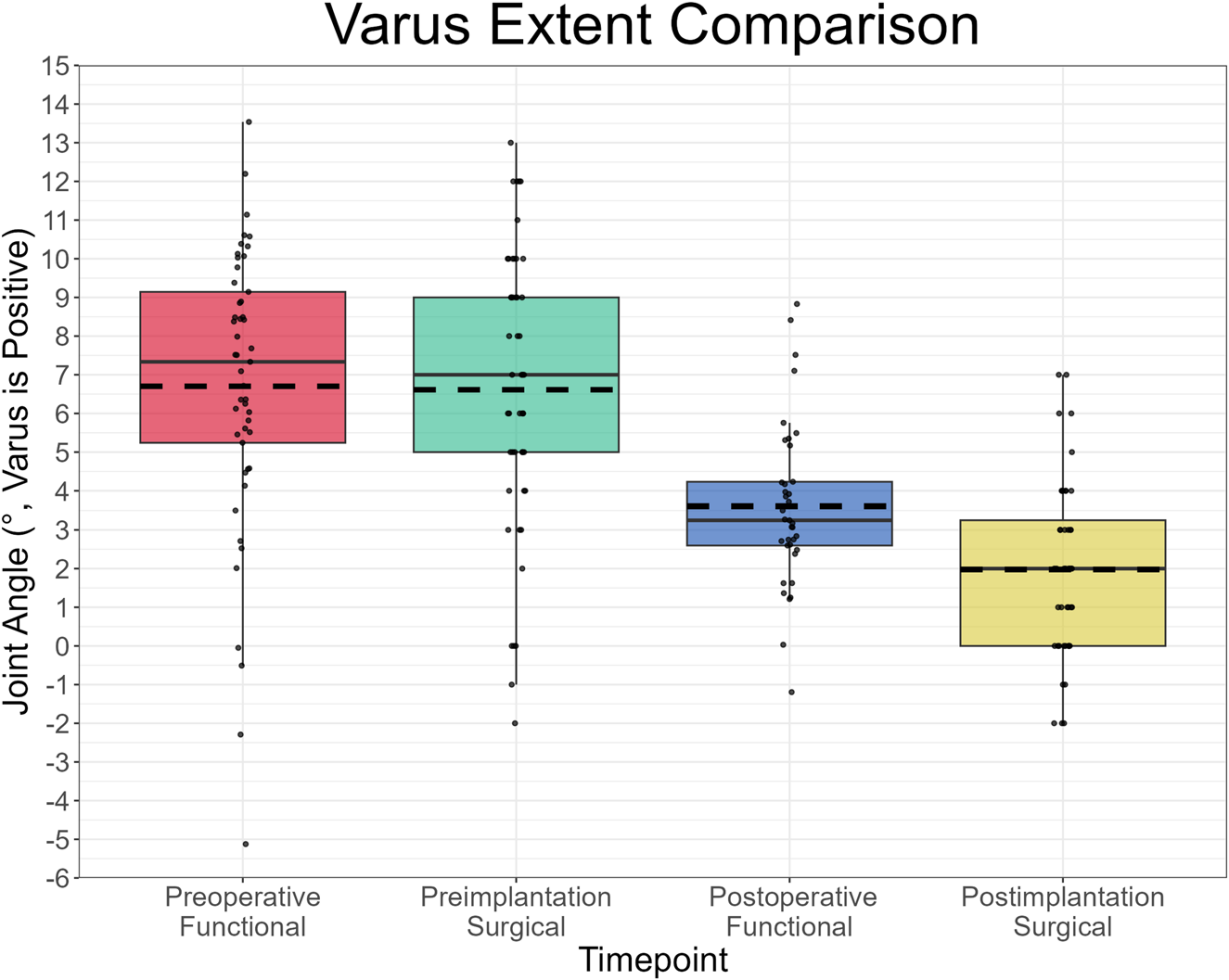
Boxplot of the resulting hip–knee–ankle (HKA) angle in the varus stress state across the preoperative, intraoperative and postoperative settings. Each pair of measurements (except preoperative functional and preimplantation surgical) was statistically significantly different to each other (*p* < .05).

**Figure 5:**
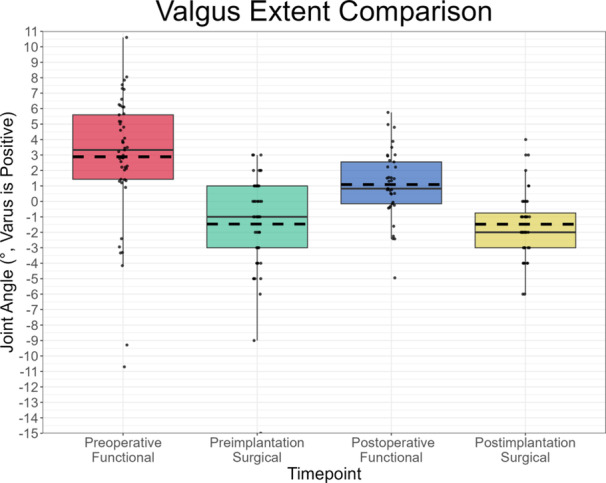
Boxplot of the resulting hip–knee–ankle (HKA) angle in the valgus stress state across the preoperative, intraoperative and postoperative settings. Each pair of measurements (except preoperative surgical and postimplantation surgical) was statistically significantly different to each other (*p* < .05).

On average, Pre‐SL (8.1°, IQR 2.0°) was considerably greater than Pre‐FL (3.8°, IQR 2.9°), while Post‐SL (3.5°, IQR 2.3°) slightly exceeded Post‐FL (2.5°, IQR 2.7°) (Figure 3). Pearson's correlation indicated a moderate relationship between Post‐SL and both Pre‐SL and Post‐FL (*r* = 0.46, *p* = .002 and *r* = 0.40, *p* = .02, respectively). Pre‐SL was greater than Pre‐FL for 92%, Post‐SL exceeded Post‐FL for 100% and Pre‐FL was greater than Post‐FL for 73% of joints with respective data available, resulting in positive mean differences in preimplantation, postimplantation, surgical and functional joint laxities (Table 3). Correctability was observed in 45 and 100% of knees preoperatively and intraoperatively (preimplantation), respectively. Varus stress yielded larger mean HKA angles preoperatively (6.7°, IQR 3.9°) and preimplantation (6.6°, IQR 4.0°) than postoperatively (3.6°, IQR 1.6°) and postimplantation (2.0°, IQR 3.3°). Mean valgus stress HKA angles were 2.9° (IQR 4.2°), −1.5° (IQR 4.0°), 1.1° (IQR 2.7°) and −1.5° (IQR 2.3°) varus for the preoperative, preimplantation, postoperative and postimplantation measurements, respectively. Figures 4 and 5 illustrate the distribution of these varus and valgus stress HKA angles, respectively.

**Table 3:** The mean (and IQR) varus and valgus stress HKA angles and joint laxity at the different timepoints and the difference in these measurements between timepoints.

	Pre			Post			Pre‐to‐post	
	Functional (*n* = 49)	Surgical (*n* = 49)	Difference (absolute) (*n* = 49)	Functional (*n* = 37)	Surgical (*n* = 44)	Difference (absolute) (*n* = 34)	Functional measurement difference (absolute) (*n* = 37)	Surgical measurement difference (absolute) (*n* = 44)
Varus stress HKA (°, Varus)	6.7 (IQR 5.2–9.1)	6.6 (IQR 4.0–9.0)	0.1 (IQR −1.1 to 1.7) (1.6, IQR 0.5–2.3)	3.6 (IQR 2.5–4.2)	2.0 (IQR 0.0–3.3)	1.8 (IQR 0.8 – 3.2) (2.4, IQR 1.1 – 3.2)	2.9 (IQR 1.4 – 4.8) (3.6, IQR 1.6–5.6)	4.6 (IQR 2.0–6.3) (4.7, IQR 2.0–6.3)
Valgus stress HKA (°, Varus)	2.9 (IQR 1.4–5.6)	−1.5 (IQR −3.0 to 1.0)	4.4 (IQR 2.8–6.0) (4.4, IQR 2.8–6.0)	1.1 (IQR 0.0–2.8)	−1.5 (IQR −3.0 to −0.8)	2.4 (IQR 1.2 – 3.5) (2.7, IQR 1.7 – 3.6)	1.5 (IQR −0.8 – 4.0) (3.6, IQR 1.6 – 4.7)	0.0 (IQR −1.0 to 2.0) (2.0, IQR 1.0–3.0)
Laxity range (°)	3.8 (IQR 2.4–5.3)	8.1 (IQR 7.0–9.0)	4.3 (IQR 2.3–7.0) (4.5, IQR 2.3–7.0)	2.5 (IQR 1.0−3.6)	3.5 (IQR 2.0–4.3)	0.7 (IQR −0.3 to 2.0) (1.5, IQR 0.5–2.1)	1.5 (IQR −0.5 to 3.6) (2.4, IQR 1.3–3.8)	4.6 (IQR 3.0–6.0) (4.6, IQR 3.0–6.0)

*Note*: A positive stress HKA value denotes a varus HKA angle.

Abbreviations: HKA, hip–knee–ankle angle; IQR, interquartile range.

The study cohort displayed significant improvements in all five KOOS subscales from the preoperative to 12‐month postoperative state (Table 4).

**Table 4:** Mean preoperative and 12‐month postoperative KOOS subdomain scores and the difference in scores between the two timepoints.

KOOS subdomain	Preoperative	Postoperative (12 months)	*p* Value	Difference
Pain	42.9 (IQR 19.4)	88.4 (IQR 11.1)	<.001	45.5 (IQR 27.8)
Symptoms	43.9 (IQR 32.1)	84.0 (IQR 14.3)	<.001	40.1 (IQR 39.3)
ADL	46.5 (IQR 25.5)	88.6 (IQR 14.1)	<.001	42.1 (IQR 36.8)
QOL	25.3 (IQR 31.3)	77.6 (IQR 31.3)	<.001	52.3 (IQR 50.0)
Sports	19.8 (IQR 31.7)	77.8 (IQR 28.3)	<.001	59.4 (IQR 50.0)

Abbreviations: ADL, activities of daily living; IQR, interquartile range; KOOS, knee injury and osteoarthritis outcome score; QOL, quality of life.

Significant correlations were observed between both Pre‐FL and preoperative valgus stress HKA angle and postoperative KOOS symptoms (*r* = 0.33, *p* = .02 and *r* = −0.28, *p* = .05, respectively) and QOL (*r* = 0.38, *p* = .01 and *r* = −0.31, *p* = .03, respectively) scores. Post‐FL correlated with improvements in KOOS ADL (*r* = 0.35, *p* = .04) scores. Although there were no significant relationships between preimplantation SL measurements and PROMS, Post‐SL displayed moderate negative correlation with postoperative KOOS Symptoms (*r* = −0.37, *p* = .01), which was driven by the varus stress HKA angle (*r* = 0.35, *p* = .02). Additionally, differences in FL and functional valgus stress HKA angles significantly correlated with postoperative KOOS QOL outcomes (*r* = 0.35, *p* = .03 and *r *= −0.41, *p* = .01, respectively). Table 5 displays these correlations with the five KOOS subscales.

**Table 5:** Correlation of stress HKA angles and laxity data to the five KOOS subdomains preoperatively and postoperatively and the change in the scores from pre‐to‐postoperative state for these subdomains.

	Postoperative (12 months)					Improvement				
	Pain	Symptoms	ADL	QOL	Sports	Pain	Symptoms	ADL	QOL	Sports
Preoperative										
Varus stress HKA	0.05	−0.07	0.04	−0.07	0.15	−0.11	−0.14	−0.05	0.00	0.10
Valgus stress HKA	−0.01	−0.28*	−0.05	−0.31*	−0.01	−0.11	−0.18	−0.12	−0.14	0.04
Joint laxity (Pre‐FL)	0.11	0.33*	0.15	0.38*	0.23	0.03	0.08	0.09	0.19	0.05
Preimplantation (intraoperative)										
Varus stress HKA	0.12	0.00	0.17	−0.16	0.28	−0.12	−0.07	0.01	−0.09	0.22
Valgus stress HKA	0.04	−0.15	−0.05	−0.23	0.01	−0.08	−0.13	0.01	−0.02	0.17
Joint laxity (Pre‐SL)	0.13	0.22	0.27	0.06	0.39*	−0.12	0.00	−0.02	−0.13	0.06
Postimplantation (intraoperative)										
Varus stress HKA	0.20	0.35*	0.15	0.15	0.16	−0.05	0.06	−0.10	−0.08	−0.27
Valgus stress HKA	−0.02	−0.23	−0.18	−0.32*	−0.14	0.22	0.24	0.20	0.25	0.13
Joint laxity (Post‐SL)	−0.18	−0.37*	−0.20	−0.25	−0.21	0.15	0.10	0.15	0.22	0.34
Postoperative										
Varus stress HKA	0.09	0.01	−0.06	−0.10	−0.15	−0.11	−0.27	−0.03	−0.18	−0.05
Valgus stress HKA	0.00	−0.05	−0.12	−0.11	−0.02	−0.27	−0.30	−0.29	−0.25	−0.13
Joint laxity (Post‐FL)	0.15	0.09	0.13	0.02	−0.20	0.24	0.15	0.35*	0.19	0.28
Difference (preimplantation)										
Varus stress HKA	−0.12	−0.13	−0.22	0.13	−0.23	0.11	0.00	−0.03	0.15	−0.04
Valgus stress HKA	−0.07	−0.22	−0.08	−0.16	0.02	−0.04	−0.10	−0.13	−0.14	−0.06
Joint laxity	0.02	−0.05	0.09	−0.17	0.14	−0.11	−0.08	−0.07	−0.20	0.01
Difference (postimplantation)										
Varus stress HKA	−0.05	0.04	0.02	0.03	−0.16	0.05	0.09	0.12	−0.05	−0.06
Valgus stress HKA	−0.15	0.04	−0.11	0.10	−0.09	0.01	0.10	−0.07	−0.02	−0.04
Joint laxity	0.00	−0.03	−0.07	0.13	0.13	−0.01	−0.08	−0.15	0.03	0.04
Difference (functional)										
Varus stress HKA	−0.19	−0.13	−0.21	−0.20	0.05	−0.08	0.02	−0.16	−0.02	0.13
Valgus stress HKA	−0.19	−0.30	−0.22	−0.41*	−0.17	−0.04	−0.05	−0.10	−0.15	0.10
Joint laxity	0.06	0.23	0.13	0.35*	0.37	−0.07	0.03	−0.04	0.15	−0.04
Difference (surgical)										
Varus stress HKA	0.07	0.00	0.20	−0.18	0.37*	−0.10	0.07	0.00	−0.11	−0.11
Valgus stress HKA	0.09	0.03	0.08	−0.04	0.13	0.06	0.13	0.07	0.18	0.39*
Joint laxity	0.07	0.05	0.21	−0.10	0.36*	−0.20	−0.06	−0.09	−0.15	−0.26

*Note*: The stress HKA angles data was measured in the preoperative, intraoperative and postoperative settings and the differences were calculated between pre‐to‐intraoperative states and pre‐to‐postoperative states.

Abbreviations: ADL, activities of daily living; HKA, hip–knee–angle; KOOS, Knee injury and osteoarthritis outcome score; Post‐FL, postoperative functional laxity; Post‐SL, postoperative surgical laxity; Pre‐FL, preoperative functional laxity; Pre‐SL, preoperative surgical laxity; QOL, quality of life.

* *p* Value < .05.

**Figure 6:**
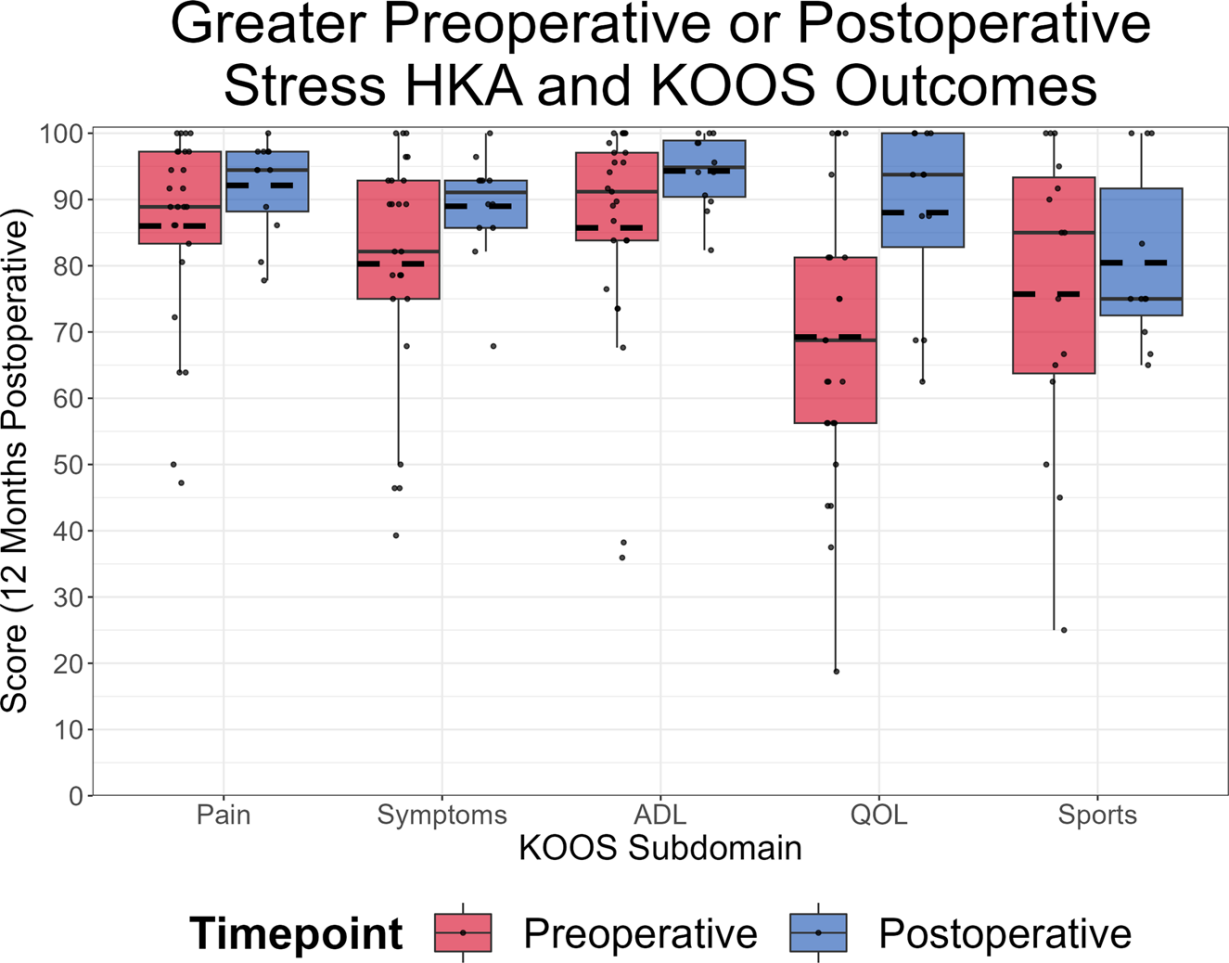
Boxplot of patient knee injury and osteoarthritis outcome score (KOOS) subdomain scores at 12 months post‐TKA based on whether patients had greater functional valgus stress hip–knee–ankle (HKA) angle preoperatively (red) or postoperatively (blue). *Denotes a statistical significance. Patients with greater functional valgus stress preoperatively than postoperatively had a mean postoperative quality of life (QOL) score of 69.2, whereas patients with a greater postoperative measurement exhibited a mean QOL score of 88.0 (*p* = .02). ADL, activities of daily living.

Patients with a more varus HKA angle during functional valgus stress postoperatively than preoperatively displayed superior KOOS QOL outcomes (Figure 6). No significant relationships were observed between greater preoperative or postoperative surgical measures and PROMs, nor between greater FL or SL or stress HKA angle measurements in the preimplantation or postimplantation settings.

## DISCUSSION

The primary finding was that Pre‐SL (8.1°, IQR 2.0°) was significantly greater than Pre‐FL (3.8°, IQR 2.9°), on average, with 92% of knees displaying greater Pre‐SL than Pre‐FL. Further, Pre‐SL deemed all knees in anaesthetised patients correctable to ±3° from neutral to the mechanical axis, while the preoperative Pre‐FL analysis in awake patients indicated only 45% as correctable. This has implications for TKA target alignment and planning as the intraoperative measurement may be excessive and overestimate the correctability of coronal deformities and knee laxity. While literature acknowledges the predictive capabilities of surgeon‐assessed intraoperative laxity for determining the magnitude of required soft‐tissue release [8, 31, 34], questions remain about whether it measures functional knee laxity [2]. Post‐SL (3.5°, IQR 2.3°) was greater than Post‐FL (2.5°, IQR 2.7°) in all joints, highlighting that the discrepancy stems from the differing laxity assessment techniques. The FL measurements involve applying controlled varus and valgus stresses to conscious patients, proven reliable for the evaluation of knee laxity and joint gaps due to the measurable and standardised force application [19, 20]. However, potential subconscious muscle contractions in awake patients, patient discomfort and tolerance to loading may limit the laxity range. Intraoperatively, SL assessment involved manual stress application to anaesthetised patients. These clinician‐applied stresses are subjective, exhibit intrasurgeon and intersurgeon variability and may be influenced by experience [4, 38].

The significantly greater mean SL difference than FL difference was driven by the preimplantation SL and FL differences. While preoperative and preimplantation varus stress HKA angles were comparable, the substantial 4.4° difference in valgus stress HKA reflects Clarke et al.'s findings of significantly greater angular displacement during intraoperative valgus stress [2]. The postimplantation laxity difference was also similar to the 0.6° implied by Clarke et al., despite their use of manual forces for the clinical laxity assessment. These findings may reflect subconscious muscle activations with valgus stress [22], potentially explaining the similar varus but different valgus stress HKA angles we observed between the preoperative and preimplantation settings.

Pre‐FL displayed significant relationships to PROMs, with correlations between Pre‐FL and postoperative KOOS QOL (*r* = 0.38) and symptoms (*r* = 0.33), suggesting that patients with greater preoperative knee laxity experienced enhanced QOL and fewer knee OA symptoms postoperatively. Conversely, negative relationships between preoperative functional valgus stress HKA angle and KOOS QOL (*r* = −0.31) and symptoms (*r* = −0.28) indicate that these superior outcomes are more prominent for joints with greater preoperative medial compartment laxity. Further research is required to understand this phenomenon, but possible explanations include greater preoperative laxity enabling easier joint correction during TKA without soft‐tissue releases, correction of preoperative laxity with balanced TKA improving joint stability and confidence and/or patients with greater laxity having more compliant tissues, achieving greater ROM.

Post‐FL was the only significant predictor of patient outcome improvement, specifically KOOS ADL (*r* = 0.35). Our study, like Hamilton et al. [9], found no relationship between postoperative laxity in awake patients and postoperative outcomes. In contrast, Mizu‐uchi et al. reported that greater postoperative valgus laxity correlated with higher postoperative symptoms and satisfaction scores [26]. These conflicting findings may stem from the use of varying PROM questionnaires. Correlations of Post‐SL and postimplantation surgical varus stress HKA measurements with KOOS Symptoms (*r* = −0.37 and *r* = 0.35, respectively) indicate that maintaining lower postimplantation laxity with some residual lateral compartment laxity enhances outcomes. This complements literature noting that residual postimplantation medial laxity or tightness negatively impacts 1‐ and 2‐year postoperative KOOS Pain [1, 43, 44], and 1‐year postoperative KOOS ADL outcomes [1]. The absence of a relationship between Pre‐SL and outcomes in our study may stem from the variability or inability to accurately quantify applied force during Pre‐SL measurement. Verasense and robotic systems enable force and displacement measurement during gap balancing and their use displays comparable short‐term outcomes to manually balanced TKAs [35, 47]. However, the application of force to anaesthetised patients may still exceed individual ligament pain tolerances. Constitutional ligament tension is likely patient‐specific, highlighting the importance of preoperative standardised assessments, such as Pre‐FL in awake patients, which provide greater control and are more indicative of patient outcomes post‐TKA. Though not always practical in common practice, they can serve as valuable research tools to better understand patient‐specific ligament properties and make TKA balancing more scientific and objective.

No significant relationships were found between preimplantation or surgical joint laxity differences and PROMs. However, a moderate correlation was observed between FL difference and postoperative KOOS QOL (*r* = 0.35), driven by the difference between preoperative and postoperative valgus stress HKA angle (*r* = −0.41). Patients with greater postoperative varus HKA angles during valgus stress, than preoperative, experienced superior postoperative KOOS QOL outcomes, implying that decreasing medial compartment laxity with TKA may enhance outcomes. These findings highlight the importance of medial compartment stability throughout knee ROM [44], given the comparative tolerance of lateral laxity [17, 42]. This might be attributed to greater native lateral laxity [29, 37] and diminishing lateral collateral ligamentous laxity post‐TKA [36], suggesting that medial tibiofemoral joint stability should be prioritised during surgical planning and technique.

### Limitations

Our study has several limitations. The stress radiology protocol requires applying a controlled force of up to 150 N or the patient's maximum threshold, potentially affecting reliability if the ideal patient‐specific force is not used. While the final applied load was not recorded, studies applying 150 N for coronal laxity assessment in osteoarthritic knees report no significant discomfort [13, 23, 26, 46]. The Telos SD‐900, effective for knee laxity research [13, 14, 15, 19, 23, 27, 42], is labour‐intensive and not commonly available in clinical practice. More scalable imaging protocols like those by Jagota et al. [16] and Tokuhara et al. [40], involving knee distraction with ankle weights, are needed for broader clinical application. The limited sample sizes of 44 and 37 out of 49 joints with postimplantation measurements and postoperative stress radiographs, respectively, were also a constraint, partly due to the time‐consuming protocol and retrospective analysis, which may impact results [39]. The surgeon's intraoperative laxity assessment technique is subjective and may vary between surgeons. However, the surgeon has 15 years of experience with a high TKA volume. Preimplantation intraoperative laxity assessments were performed after medial arthrotomy but before anterior cruciate ligament or osteophyte removal, and ligament release, which may have contributed to the variations between preoperative and intraoperative laxities due to soft‐tissue release during exposure. Additionally, Pre‐FL measurements may not accurately reflect the patient's constitutional laxity due to the diseased state of the knee. Nevertheless, we observed greater correlations between PROMs and awake Pre‐FL than anaesthetised Pre‐SL.

## CONCLUSIONS

Preoperative assessment of coronal knee laxity in an awake patient is considerably different from intraoperative laxity measurements with patients under anaesthesia. While preoperative laxity assessment better predicts postoperative patient outcomes and is arguably a more suitable measurement for the native and functional coronal knee laxity, further research is required to understand the factors contributing to this relationship. The stress radiology protocol used for preoperative and postoperative laxity assessment enables consistent comparison of laxity before and after TKA and allows for soft‐tissue‐informed preoperative planning.

## AUTHOR CONTRIBUTIONS

**Ishaan Jagota**: Conceptualisation; formal analysis; investigation; methodology; project administration; visualisation; writing—original draft. **Rami M. A. Al‐Dirini**: Supervision; writing—review and editing. **Mark Taylo**r: Supervision; writing—review and editing. **Joshua Twiggs**: Conceptualisation; methodology; supervision; writing—review and editing. **Brad Miles**: Conceptualisation; resources; supervision; writing—review and editing. **David Liu**: Conceptualisation; methodology; data curation; supervision; writing—review and editing.

## CONFLICTS OF INTEREST STATEMENT

At the time of writing this manuscript, Ishaan Jagota, Joshua Twiggs and Brad Miles were all employees of 360 Med Care, Mathys Orthopaedics and Enovis ANZ. This research was supported by 360 Med Care, Mathys Orthopaedics and Enovis Australia. Brad Miles has stock or stock options in Enovis and 360 Med Care, has/does receive royalties from Enovis ANZ and Corin, and has been a paid consultant for 360 Med Care, Corin and Johnson & Johnson. David Liu receives royalties from Zimmer Biomet, is a paid spreaker for Zimmer Biomet, Depuy and LifeHealthCare. Rami M. A. Al‐Dirini and Mark Taylor have directly or indirectly received research support from 360 Med Care. David Liu is on the editorial boards of *Knee Surgery and Related Research and Arthroplasty*. David Liu is also a board/committee member of Asia Pacific Arthroplasty Society and Knee Arthroplasty Subcommittee.

## ETHICS STATEMENT

Ethical approval was obtained from the Bellberry Human Research Ethics Committee (study number 201203710). This study was performed in line with the principles of the 1964 Helsinki Declaration. Patient consent was obtained to use their patient and clinical data for research activities.

## Supporting information

Supporting information.

## References

[1] Aunan, E. , Kibsgård, T.J. , Diep, L.M. & Röhrl, S.M. (2015) Intraoperative ligament laxity influences functional outcome 1 year after total knee arthroplasty. Knee Surgery, Sports Traumatology, Arthroscopy, 23(6), 1684–1692. Available from: 10.1007/s00167-014-3108-0 PMC4439434 24917538

[2] Clarke, J.V. , Deakin, A.H. , Picard, F. & Riches, P.E. (2017) Lower limb alignment and laxity measures before, during and after total knee arthroplasty: a prospective cohort study. Clinical Biomechanics, 47, 61–65. Available from: 10.1016/j.clinbiomech.2017.05.013 28600996

[3] Cretu, B. , Costache, M. , Cursaru, A. , Serban, B. , Spiridonica, R. , Popa, M. et al. (2023) Restoring anatomical features in primary total knee arthroplasty. Cureus. 15(6), e40616. Available from: 10.7759/cureus.40616 37342300 PMC10278159

[4] Elmasry, S.S. , Sculco, P.K. , Kahlenberg, C.A. , Mayman, D.J. , Cross, M.B. , Pearle, A.D. et al. (2022) Arthroplasty surgeons differ in their intraoperative soft tissue assessments: a study in human cadavers to quantify surgical decision‐making in TKA. Clinical Orthopaedics & Related Research, 480(8), 1604–1615. Available from: 10.1097/CORR.0000000000002184 35323146 PMC9278950

[5] Fürmetz, J. , Sass, J. , Ferreira, T. , Jalali, J. , Kovacs, L. , Mück, F. et al. (2019) Three‐dimensional assessment of lower limb alignment: accuracy and reliability. The Knee, 26(1), 185–193. Available from: 10.1016/j.knee.2018.10.011 30473372

[6] Graichen, H. , Lekkreusuwan, K. , Eller, K. , Grau, T. , Hirschmann, M.T. & Scior, W. (2022) A single type of varus knee does not exist: morphotyping and gap analysis in varus OA. Knee Surgery, Sports Traumatology, Arthroscopy, 30(8), 2600–2608. Available from: 10.1007/s00167-021-06688-4 34414473

[7] Grosso, M.J. , Wakelin, E.A. , Plaskos, C. & Lee, G.C. (2024) Alignment is only part of the equation: high variability in soft tissue distractibility in the varus knee undergoing primary TKA. Knee Surgery, Sports Traumatology, Arthroscopy, 32(6), 1516–1524. Available from: 10.1002/ksa.12115 38488243

[8] Hakki, S. , Coleman, S. , Saleh, K. , Bilotta, V.J. & Hakki, A. (2009) Navigational predictors in determining the necessity for collateral ligament release in total knee replacement. The Journal of Bone and Joint Surgery. British Volume, 91–B(9), 1178–1182. Available from: 10.1302/0301-620X.91B9.22043 19721043

[9] Hamilton, D.F. , Mandziak, D. , Sehgal, A. , Howie, C.R. & Burnett, R. (2020) Variation in ligamentous laxity in well‐functioning total knee arthroplasty is not associated with clinical outcomes or functional ability. European Journal of Orthopaedic Surgery & Traumatology, 30(5), 827–833. Available from: 10.1007/s00590-020-02634-1 32025865 PMC7300098

[10] Hess, S. , Moser, L.B. , Robertson, E.L. , Behrend, H. , Amsler, F. , Iordache, E. et al. (2022) Osteoarthritic and non‐osteoarthritic patients show comparable coronal knee joint line orientations in a cross‐sectional study based on 3D reconstructed CT images. Knee Surgery, Sports Traumatology, Arthroscopy, 30(2), 407–418. Available from: 10.1007/s00167-021-06740-3 PMC8866364 34564737

[11] Hirschmann, M.T. , Moser, L.B. , Amsler, F. , Behrend, H. , Leclerq, V. & Hess, S. (2019) Functional knee phenotypes: a novel classification for phenotyping the coronal lower limb alignment based on the native alignment in young non‐osteoarthritic patients. Knee Surgery, Sports Traumatology, Arthroscopy, 27(5), 1394–1402. Available from: 10.1007/s00167-019-05509-z 30976825

[12] Holland, C.T. , Savov, P. , Ettinger, M. & Seyler, T.M. (2024) Defining distinct stress curve morphologies for coronal plane alignment of the knee phenotypes using an imageless navigation robotic platform in total knee arthroplasty. The Journal of Arthroplasty. Available from: 10.1016/j.arth.2024.06.011 38879091

[13] Ishii, Y. , Matsuda, Y. , Ishii, R. , Sakata, S. & Omori, G. (2003) Coronal laxity in extension in vivo after total knee arthroplasty. Journal of Orthopaedic Science, 8(4), 538–542. Available from: 10.1007/s00776-003-0668-0 12898307

[14] Ishii, Y. , Noguchi, H. , Matsuda, Y. , Kiga, H. , Takeda, M. & Toyabe, S. (2009) Preoperative laxity in osteoarthritis patients undergoing total knee arthroplasty. International Orthopaedics, 33(1), 105–109. Available from: 10.1007/s00264-007-0467-x 17938923 PMC2899222

[15] Ishii, Y. , Noguchi, H. , Sato, J. , Ishii, H. & Toyabe, S. (2019) Mediolateral coronal laxity does not correlate with knee range of motion after total knee arthroplasty. Archives of Orthopaedic and Trauma Surgery, 139(6), 851–858. Available from: 10.1007/s00402-019-03161-3 30859302

[16] Jagota, I. , Twiggs, J. , Miles, B. & Liu, D. (2024) Preoperative joint distraction imaging and planning protocol for total knee arthroplasty. The Journal of Arthroplasty, 39, 1259–1265. Available from: 10.1016/j.arth.2023.11.025 38007203

[17] Kamenaga, T. , Muratsu, H. , Kanda, Y. , Miya, H. , Kuroda, R. & Matsumoto, T. (2018) The influence of postoperative knee stability on patient satisfaction in cruciate‐retaining total knee arthroplasty. The Journal of Arthroplasty, 33(8), 2475–2479. Available from: 10.1016/j.arth.2018.03.017 29656976

[18] Kappel, A. , Laursen, M. , Nielsen, P.T. & Odgaard, A. (2019) Relationship between outcome scores and knee laxity following total knee arthroplasty: a systematic review. Acta Orthopaedica, 90(1), 46–52. Available from: 10.1080/17453674.2018.1554400 30569797 PMC6367957

[19] Kappel, A. , Mortensen, J.F. , Nielsen, P.T. , Odgaard, A. & Laursen, M. (2020) Reliability of stress radiography in the assessment of coronal laxity following total knee arthroplasty. The Knee, 27(1), 221–228. Available from: 10.1016/j.knee.2019.09.013 31875838

[20] Koppens, D. , Sørensen, O.G. , Munk, S. , Rytter, S. , Larsen, S.K.A. , Stilling, M. et al. (2019) The lateral joint space width can be measured reliably with Telos valgus stress radiography in medial knee osteoarthritis. Skeletal Radiology, 48(7), 1069–1077. Available from: 10.1007/s00256-018-3111-5 30456552

[21] Lee, O.‐S. , Elazab, A. & Lee, Y.S. (2019) Preoperative varus‐valgus stress angle difference is valuable for predicting the extent of medial release in varus deformity during total knee arthroplasty. Knee Surgery and Related Research, 31(1), 12–18. Available from: 10.5792/ksrr.18.033 PMC6425900 30871287

[22] Lewek, M.D. , Ramsey, D.K. , Snyder‐Mackler, L. & Rudolph, K.S. (2005) Knee stabilization in patients with medial compartment knee osteoarthritis. Arthritis & Rheumatism, 52(9), 2845–2853. Available from: 10.1002/art.21237 16142714 PMC1343471

[23] Li, J. , Liu, D. , Baré, J. , Dickison, D. , Theodore, W. , Miles, B. et al. (2022) Correctability of the knee joint observed under a stressed state. The Knee, 34, 206–216. Available from: 10.1016/j.knee.2021.12.004 34992024

[24] MacDessi, S.J. , Gharaibeh, M.A. & Harris, I.A. (2019) How accurately can soft tissue balance be determined in total knee arthroplasty? The Journal of Arthroplasty, 34(2), 290–294.e1. Available from: 10.1016/j.arth.2018.10.003 30389257

[25] MacDessi, S.J. , Griffiths‐Jones, W. , Harris, I.A. , Bellemans, J. & Chen, D.B. (2021) Coronal plane alignment of the knee (CPAK) classification: a new system for describing knee phenotypes. The Bone & Joint Journal, 103–B(2), 329–337. Available from: 10.1302/0301-620X.103B2.BJJ-2020-1050.R1 PMC7954147 33517740

[26] Mizu‐uchi, H. , Kawahara, S. , Ishibashi, S. , Colwell, C.W. , Nakashima, Y. & D'Lima, D.D. (2022) Postoperative valgus laxity and medial pivot kinematics are significantly associated with better clinical outcomes. The Journal of Arthroplasty, 37(6), S187–S192. Available from: 10.1016/j.arth.2022.02.088 35231562

[27] Nakahara, H. , Okazaki, K. , Hamai, S. , Okamoto, S. , Kuwashima, U. , Higaki, H. et al. (2015) Does knee stability in the coronal plane in extension affect function and outcome after total knee arthroplasty? Knee Surgery, Sports Traumatology, Arthroscopy, 23(6), 1693–1698. Available from: 10.1007/s00167-014-3122-2 24923689

[28] Okamoto, N. , Nakamura, E. , Masuda, T. , Hisanaga, S. & Miyamoto, T. (2024) Lateral laxity in flexion influences patient‐reported outcome after total knee arthroplasty. Indian Journal of Orthopaedics, 58(1), 24–29. Available from: 10.1007/s43465-023-01045-8 38161401 PMC10754782

[29] Okazaki, K. , Miura, H. , Matsuda, S. , Takeuchi, N. , Mawatari, T. , Hashizume, M. et al. (2006) Asymmetry of mediolateral laxity of the normal knee. Journal of Orthopaedic Science, 11(3), 264–266. Available from: 10.1007/s00776-006-1009-x 16721527

[30] Oussedik, S. , Abdel, M.P. , Victor, J. , Pagnano, M.W. & Haddad, F.S. (2020) Alignment in total knee arthroplasty. The Bone & Joint Journal, 102–B(3), 276–279. Available from: 10.1302/0301-620X.102B3.BJJ-2019-1729 32114811

[31] Picard, F. , Deakin, A.H. , Clarke, J.V. , Dillon, J.M. & Gregori, A. (2007) Using navigation intraoperative measurements narrows range of outcomes in TKA. Clinical Orthopaedics & Related Research, 463, 50–57. Available from: 10.1097/BLO.0b013e3181468734 17632421

[32] Sabatini, L. , Barberis, L. , Camazzola, D. , Centola, M. , Capella, M. , Bistolfi, A. et al. (2021) Bicruciate‐retaining total knee arthroplasty: what's new? World Journal of Orthopedics, 12(10), 732–742. Available from: 10.5312/wjo.v12.i10.732 34754829 PMC8554348

[33] Salvadore, G. , Meere, P.A. , Verstraete, M.A. , Victor, J. & Walker, P.S. (2018) Laxity and contact forces of total knee designed for anatomic motion: a cadaveric study. The Knee, 25(4), 650–656. Available from: 10.1016/j.knee.2018.04.014 29778656

[34] Saragaglia, D. , Chaussard, C. & Rubens‐Duval, B. (2006) Navigation as a predictor of soft tissue release during 90 cases of computer‐assisted total knee arthroplasty. Orthopedics, 29(10 supplement), 137–138. 17407940

[35] Sava, M.‐P. , Hara, H. , Alexandra, L. , Hügli, R.W. & Hirschmann, M.T. (2023) Verasense sensor‐assisted total knee arthroplasty showed no difference in range of motion, reoperation rate or functional outcomes when compared to manually balanced total knee arthroplasty: a systematic review. Knee Surgery, Sports Traumatology, Arthroscopy, 31(5), 1851–1858. Available from: 10.1007/s00167-023-07352-9 PMC10090011 36854996

[36] Sekiya, H. , Takatoku, K. , Takada, H. , Sasanuma, H. & Sugimoto, N. (2009) Postoperative lateral ligamentous laxity diminishes with time after TKA in the varus knee. Clinical Orthopaedics and Related Research®, 467(6), 1582–1586. Available from: 10.1007/s11999-008-0588-6 18941848 PMC2674159

[37] Shalhoub, S. , Moschetti, W.E. , Dabuzhsky, L. , Jevsevar, D.S. , Keggi, J.M. & Plaskos, C. (2018) Laxity profiles in the native and replaced knee—application to robotic‐assisted gap‐balancing total knee arthroplasty. The Journal of Arthroplasty, 33(9), 3043–3048. Available from: 10.1016/j.arth.2018.05.012 29909956

[38] Sohmiya, K. , Ogawa, H. , Nakamura, Y. , Sengoku, M. , Shimokawa, T. , Ohnishi, K. et al. (2023) Joint gap produced by manual stress is dependent on the surgeon's experience and is smaller in flexion in robotic‐assisted total knee arthroplasty. Knee Surgery, Sports Traumatology, Arthroscopy, 31(3), 963–968. Available from: 10.1007/s00167-022-07107-y 35969256

[39] Talari, K. & Goyal, M. (2020) Retrospective studies—utility and caveats. Journal of the Royal College of Physicians of Edinburgh, 50(4), 398–402. Available from: 10.4997/jrcpe.2020.409 33469615

[40] Tokuhara, Y. , Kadoya, Y. , Kanekasu, K. , Kondo, M. , Kobayashi, A. & Takaoka, K. (2006) Evaluation of the flexion gap by axial radiography of the distal femur. The Journal of Bone and Joint Surgery. British Volume, 88–B(10), 1327–1330. Available from: 10.1302/0301-620X.88B10.17793 17012422

[41] Tsukeoka, T. & Tsuneizumi, Y. (2016) Varus and valgus stress tests after total knee arthroplasty with and without anesthesia. Archives of Orthopaedic and Trauma Surgery, 136(3), 407–411. Available from: 10.1007/s00402-015-2405-5 26742494

[42] Tsukiyama, H. , Kuriyama, S. , Kobayashi, M. , Nakamura, S. , Furu, M. , Ito, H. et al. (2017) Medial rather than lateral knee instability correlates with inferior patient satisfaction and knee function after total knee arthroplasty. The Knee, 24(6), 1478–1484. Available from: 10.1016/j.knee.2017.09.004 28970125

[43] Wakelin, E.A. , Ponder, C.E. , Randall, A.L. , Koenig, J.A. , Plaskos, C. , DeClaire, J.H. et al. (2023) Intra‐operative laxity and balance impact 2‐year pain outcomes in TKA: a prospective cohort study. Knee Surgery, Sports Traumatology, Arthroscopy, 31(12), 5535–5545. Available from: 10.1007/s00167-023-07601-x 37837574

[44] Wakelin, E.A. , Shalhoub, S. , Lawrence, J.M. , Keggi, J.M. , DeClaire, J.H. , Randall, A.L. et al. (2022) Improved total knee arthroplasty pain outcome when joint gap targets are achieved throughout flexion. Knee Surgery, Sports Traumatology, Arthroscopy, 30(3), 939–947. Available from: 10.1007/s00167-021-06482-2 33580346

[45] Wakelin, E.A. , Tran, L. , Twiggs, J.G. , Theodore, W. , Roe, J.P. , Solomon, M.I. et al. (2018) Accurate determination of post‐operative 3D component positioning in total knee arthroplasty: the AURORA protocol. Journal of Orthopaedic Surgery and Research, 13(1), 275. Available from: 10.1186/s13018-018-0957-0 30376865 PMC6208069

[46] Waldstein, W. , Monsef, J.B. , Buckup, J. & Boettner, F. (2013) The value of valgus stress radiographs in the workup for medial unicompartmental arthritis. Clinical Orthopaedics & Related Research, 471(12), 3998–4003. Available from: 10.1007/s11999-013-3212-3 23917994 PMC3825882

[47] Zhang, J. , Ndou, W.S. , Ng, N. , Gaston, P. , Simpson, P.M. , Macpherson, G.J. et al. (2022) Robotic‐arm assisted total knee arthroplasty is associated with improved accuracy and patient reported outcomes: a systematic review and meta‐analysis. Knee Surgery, Sports Traumatology, Arthroscopy, 30(8), 2677–2695. Available from: 10.1007/s00167-021-06464-4 PMC9309123 33547914

[48] Zhao, R. , Liu, Y. & Tian, H. (2021) Accuracy of soft tissue balancing in total knee arthroplasty using surgeon‐defined assessment versus a gap‐balancer or electronic sensor. Journal of Orthopaedic Surgery and Research, 16(1), 305. Available from: 10.1186/s13018-021-02439-w 33964958 PMC8106209

