# Supplementary material for: Functional preoperative assessment of coronal knee laxity better predicts postoperative patient outcomes than intraoperative surgeon‐defined laxity in total knee arthroplasty
Source: Knee Surg Sports Traumatol Arthrosc. 2024 Sep 3;33(2):621–33. doi: 10.1002/ksa.12400 (PMC11792101; doi:10.1002/ksa.12400)
Supplement: Supplementary file 1 — Supporting information. [file KSA-33-621-s001.docx]

Appendix

Appendix A. Descriptions of landmarks identified during image processing. PCL, posterior cruciate ligament; CT, computed tomography.

| Landmark | Bone | Description |
| --- | --- | --- |
| Femoral Head Centre | Femur | The hip centre, marking the centre of the femoral head at the hip joint. |
| Distal Femoral Centre | Femur | The centre of the intercondylar femoral notch. |
| Lateral Distal Condyle | Femur | The most distal point on the lateral femoral condyle. |
| Medial Distal Condyle | Femur | The most distal point on the medial femoral condyle. |
| Lateral Epicondyle | Femur | The most lateral point on the femur's curved ridge, which is approximately equidistant from the posterior and distal surfaces of the lateral condyle. |
| Medial Sulcus | Femur | Located in the depression on the femur's medial side. |
| Medial Malleolus | Tibia | The most prominent point on the medial side of the distal tibia. |
| Lateral Malleolus | Fibula | The most lateral point on the distal fibular ridge. |
| Ankle Centre | Tibia | The midpoint of the medial and lateral malleoli landmarks. |
| Tubercle | Tibia | The medial third supero-inferior line of the prominence on the tibia's anterior face. |
| PCL Insertion | Tibia | The most posterior prominence on the tibia, midway between the condylar plateaus. |
| Tibial Centre | Tibia | The midpoint of the tubercle and PCL insertion landmarks. |
